# Potential role of inflammation in relation to dietary sodium and β-carotene with non-alcoholic fatty liver disease: a mediation analysis

**DOI:** 10.1038/s41387-022-00218-y

**Published:** 2022-09-15

**Authors:** Yang Chen, Min Wu, Fuli Chen, Xiaoxiao Wen, Liancheng Zhao, Gang Li, Long Zhou

**Affiliations:** 1 Department of Cardiology, Sichuan Provincial People’s Hospital, University of Electronic Science and Technology of China, Chengdu, China; 2 School of Medicine, Sichuan Provincial People’s Hospital, University of Electronic Science and Technology of China, Chengdu, China; 3 Department of Epidemiology, College of Public Health and Health Professions and College of Medicine, University of Florida, Gainesville, FL USA; 4 Division of Prevention and Community Health, National Center for Cardiovascular Disease, Fuwai Hospital, Chinese Academy of Medical Sciences and Peking Union Medical College, Beijing, China

**Keywords:** Risk factors, Metabolic syndrome

## Abstract

**Background:**

High sodium intake has been linked to the prevalence of non-alcoholic fatty liver disease (NAFLD), but underlying mechanism remains unclear. This study aims to explore the role of chronic inflammation in the association between sodium and NAFLD. We also observed whether β-carotene, which had a strong anti-inflammatory effect, lowers the odds of NAFLD.

**Methods:**

We performed mediation analyses to assess the mediating effects of C-reactive protein (CRP) and red cell distribution width (RDW) on the relationship between dietary sodium and NAFLD defined by the hepatic steatosis index (HSI) and the fatty liver index (FLI), respectively.

**Results:**

A total of 6725 participants were included in this study. Compared with the high sodium-low carotene group, participants in the high sodium-high carotene group had 16% and 26% lower odds for HSI and FLI-defined NAFLD, respectively. There were positive indirect effects of dietary sodium intake on the HSI-defined NAFLD (indirect effect: 0.0057, 95% CI: 0.0021–0.0091, *P* < 0.0001), as well as the FLI defined NAFLD (indirect effect: 0.0081, 95% CI: 0.0024–0.0162, *P* < 0.0001) when C-reactive protein (CRP) was considered as a mediator. The mediating effects were somewhat attenuated after further adjusting for dietary β-carotene intake. Similar results were found when RDW was considered as a mediator in the HSI-defined NAFLD analysis.

**Conclusions:**

Higher sodium intake increases the odds of NAFLD by upregulating inflammation. Dietary β-carotene may attenuate this association by down regulating inflammation.

## Introduction

High sodium intake has been confirmed as one of the most important risk factors for high blood pressure [1–3]. Excessive sodium intake also has been linked to higher risk of overweight/obesity as well as cardiovascular diseases [4–7]. Our previous study reported that dietary sodium intake was positively associated with non-alcoholic fatty liver disease (NAFLD) in a representative US general population [8]. NAFLD is a liver condition defined as the accumulation of intracellular fat in hepatocytes in the absence of excessive alcohol intake, and it is considered the hepatic expression of the metabolic syndrome. To date, the mechanisms underlying sodium intake in relation to NAFLD remain unclear. As components of metabolic syndrome, NAFLD, high blood pressure, and overweight/obesity share a common pathophysiology, that is, chronic inflammation [9–11]. As a systemic inflammation biomarker, C-reactive protein (CRP) has been linked to disease and mortality risk [9]. Recently, red cell distribution width (RDW) has also been suggested as a marker that reflect an underlying chronic inflammatory state [12–14]. We assume that high sodium intake increases the risk of NAFLD by upregulating inflammation. If this hypothesis is true, down regulating inflammation will improve hepatic steatosis caused by high sodium intake. β-carotene, a retinol precursor and antioxidant that is found in certain fruits and vegetables, has been found to have a strong anti-inflammatory effect [15–17]. Our second hypothesis, accordingly, is that high dietary β-carotene intake attenuates the effects of high sodium intake on NAFLD by down regulating inflammation. The present study aims to use mediation analyses to test the above two hypotheses by utilizing data from two cycles (cycle 2007–2008 and cycle 2009–2010) of the National Health and Nutrition Examination Survey (NHANES).

## Subjects and methods

### Study population

**Fig. 1:**
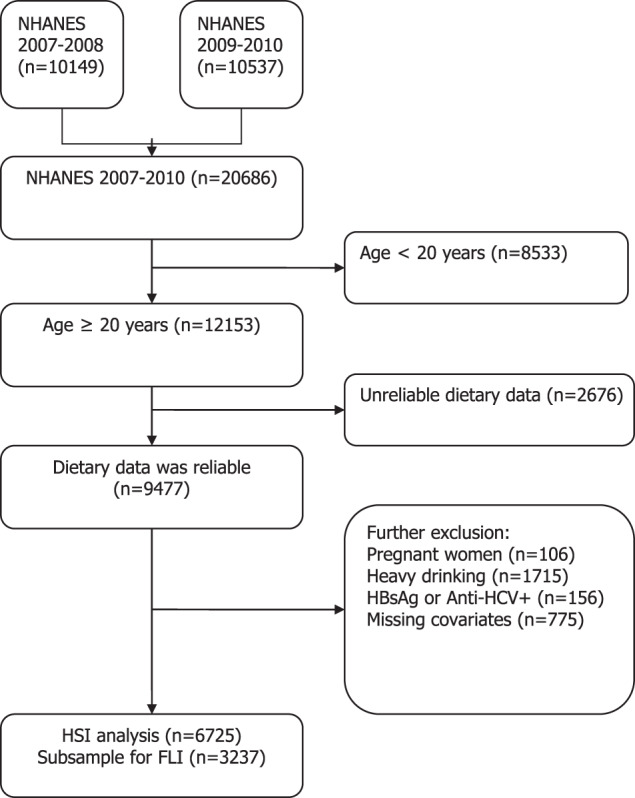
Flowchart of the study participants selection. A total of 6725 and 3237 participants were selected from the two cycles of National Health and Nutrition Examination Survey (NHANES) for hepatic steatosis index (HSI) and fatty liver index (FLI) analyses.

Data were derived from two cycles of NHANES (2007–2008 and 2009–2010). A detailed description of the NHANES study design and methods is available elsewhere [18]. In total, 20,686 individuals completed the NHANES surveys between 2007 and 2010 within two cycles (10,149 in NHANES 2007–2008 and 10,537 in NHANES 2009–2010). In this study, we included individuals aged ≥20 years who completed two 24-h recalls with valid energy intakes (*n* = 9477). A valid level was defined as total energy intake from both 24-h recalls within 500–5000 kcal/day for women and 500–8000 kcal/day for men [19]. We further excluded 2719 subjects for the following reasons: (1) pregnant women (*n* = 106); (2) heavy alcohol drinking defined as >21 drinks per week for men and >14 drinks per week for women (*n* = 1715) [8]; (3) serum hepatitis B surface antigen (HBsAg) or serum hepatitis C antibody was positive (*n* = 156); (4) important covariates were not available (*n* = 775). This resulted in 6725 participants included in the hepatic steatosis index (HSI) defined NAFLD analysis. A subsample of 3237 participants was included in the fatty liver index (FLI) defined NAFLD analysis because FLI requires fasting blood tests. A detailed flowchart depicting participant selection is shown in Fig. 1. The National Center for Health Statistics institutional review board approved the study protocol (#2005–06), and all participants provided written informed consent. All data used in this manuscript are de-identified and freely available to the public through https://wwwn.cdc.gov/nchs/nhanes/.

### Data collection

Data were collected by trained interviewers during interviews in the participants’ homes and through medical examination and subsequent laboratory assessments in the Mobile Examination Center (MEC). The questionnaire contains a number of questions on demographics, health conditions, and lifestyles such as smoking, drinking, house income, and passive sedentary behavior.

The ethnicity was divided into Hispanic, non-Hispanic White, non-Hispanic Black, and other races. As the house income variable in our analysis, family monthly poverty level index was calculated by dividing family income by the poverty guidelines, specific to family size, as well as the appropriate year and state. The index was grouped into three categories (≤1.30, 1.31–1.85, and >1.85).

According to participants’ answers to the questionnaire, smoking status was categorized into current, former, and never smoker. Drinkers were defined as participants drinking alcohol at least 12 times in the previous year. Sedentary time, defined as total time spent sitting in a typical day not including time spent sleeping, was used as a passive sedentary behavior indicator in our analysis.

Dietary intake was documented and validated utilizing the 24-h dietary history interview. Participants received two 24-h dietary recall interviews. The first dietary recall interview was collected face-to-face in the MEC, and the second was collected by telephone 3 to 10 days later. Dietary intake was estimated by the mean of the two dietary recalls. Dietary sodium (in mg/d) and β-carotene (in µg/day) intakes were used as the main exposure variables. We calculated the dietary inflammatory index (DII) score according to the calculating methods provided by a previous study [20]. DII is a literature-derived dietary tool for measuring individual dietary inflammatory potential. The DII score was used as a covariates in the analysis. Body weight (in kg) was measured using a digital weight scale and standing height (cm) was measured using a stadiometer. Body mass index (BMI) was calculated as weight in kilograms divided by height in meters squared. Waist circumference (WC) was measured to the nearest 0.1 cm at the end of the participant’s normal expiration using a tape by the standard method.

### Laboratory tests

Blood specimens were processed, stored, and shipped to Fairview Medical Center Laboratory at the University of Minnesota, Minneapolis, Minnesota for analysis. Serum alanine aminotransferase (ALT) was measured by a kinetic rate method in Beckman Coulter UniCel^®^ DxC800 Synchron Clinical System. Serum aspartate aminotransferase (AST) and gamma-glutamyl transferase (GGT) were measured by enzymatic rate methods in Beckman Coulter UniCel^®^ DxC800 Synchron Clinical System. Hemoglobin A1c (HbA1c) was measured by high-performance liquid chromatography (HPLC). The analyzer dilutes the whole blood specimen with Hemolysis & Wash Solution, and then injects a small volume of the treated specimen onto the HPLC analytical column. Separation is achieved by utilizing differences in ionic interactions between the cation exchange group on the column resin surface and the hemoglobin components. The separated hemoglobin components pass through the photometer flow cell where the analyzer measures changes in absorbance at 415 nm. The analyzer integrates and reduces the raw data, and then calculates the relative percentages of each hemoglobin fraction. We defined diabetes as HbA1c ≥ 6.5% and/or current treatment with a hypoglycemic agent or insulin [21]. The laboratory method used for triglycerides (TG) measurement was enzymatic assay using Roche/Hitachi Modular P Chemistry Analyzer. CRP was measured by latex-enhanced nephelometry on a Behring Nephelometer. Particle-enhanced assays are based on the reaction between a soluble analyte and the corresponding antigen or antibody bound to polystyrene particles. For the quantification of CRP, particles consisting of a polystyrene core and a hydrophilic shell are used in order to link anti-CRP antibodies covalently. A dilute solution of test sample is mixed with latex particles coated with mouse monoclonal anti-CRP antibodies. CRP concentrations are calculated by using a calibration curve. Data reduction of the signals is performed by using a storable logit-log function for the calibration curve. These assays are performed on a Siemens/Behring Nephelometer for quantitative CRP determination. Red cell distribution width represents the size distribution spread of the erythrocyte population derived from the red blood cell (RBC) histogram. It is the coefficient of variation, expressed in percent, of the RBC size distribution. RBC was directly measured on the Coulter® HMX. A detailed description of laboratory methods and materials used in the tests is available at https://wwwn.cdc.gov/nchs/nhanes/continuousnhanes/labmethods.aspx?BeginYear=2007.

### Definition of NAFLD

NAFLD was defined using HSI and FLI, both of which have been validated externally and used in epidemiologic studies, in the absence of other causes of chronic liver disease [22–24]. HSI and FLI were calculated using the following two formulas:

(1) HSI = 8 × (ALT/AST ratio) + BMI (+2, if female; +2, if diabetes). In this case, NAFLD was defined as HSI > 36.

(2) FLI = [*e*^0.953✕loge (TG) + 0.139✕BMI + 0.718✕loge (GGT) + 0.053✕WC − 15.745^]/[1 + *e*^0.953✕loge (TG) + 0.139✕BMI + 0.718✕loge (GGT) + 0.053✕WC – 15.745^] ✕ 100. In this case, NAFLD was defined as FLI ≥ 60.

### Statistical analysis

Data were presented as mean ± standard deviation (SD) for continuous variables and as frequency (percentage) for categorical variables. Dietary sodium and β-carotene intake were dichotomized using their median values and were cross combined into four groups (Low sodium-High carotene, Low sodium-Low carotene, High sodium-High carotene, and High sodium-Low carotene). We tested differences in baseline characteristics among the above four groups with one-way analysis of variance for continuous variables and Chi-square tests for categorical variables. The odds ratios (ORs) and 95% confidence intervals (CIs) for NAFLD of each group were estimated using unconditional binary logistic regression models. Potential covariates such as age, sex, ethnicity, education level, family monthly poverty level, passive sedentary hours, smoking status, drinking status, total energy intake, DII, and serum creatinine were included in multivariable analyses. The selection of covariates was based on clinical knowledge but not specific statistical way (e.g., forward or backward). The restricted cubic spline model was used for the dose–response analysis. When we analyzing the dose–response relationship between sodium or β-carotene and NAFLD separately, we mutually adjusted for β-carotene and sodium in the model.

**Fig. 2:**
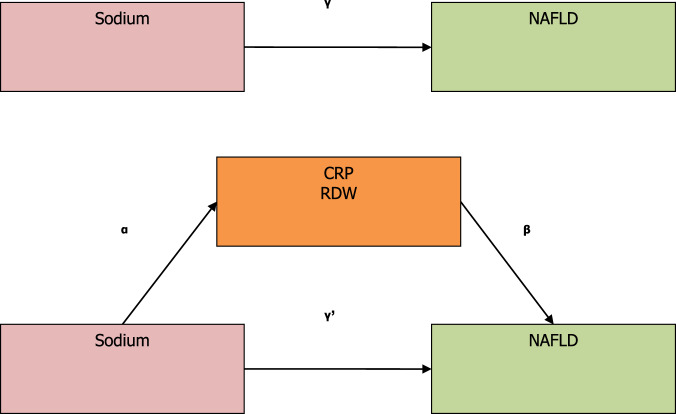
Mediation model for the associations of dietary sodium intake with no-alcoholic fatty liver disease (NAFLD) with C-reactive protein (CRP) and red cell distribution width (RDW) as the mediators. Note: Path *α* represents the regression coefficient for the association of dietary sodium intake with CRP or RDW. Path *β* represents the regression coefficient for the association of CRP or RDW with NAFLD. The product of regression coefficients *α* and *β* represents the mediated effect (indirect effect) of CRP or RDW (α*β). Path *γ*’ represents the direct effect of dietary sodium intake with NAFLD, after adjustment for CRP or RDW. Path *γ* represents the simple total effect of dietary sodium intake on NAFLD, without adjustment for CRP and RDW.

When performing mediation analysis, we followed the AGReMA statement, a guideline for reporting mediation analyses [25]. The variables of sodium intake, CRP, and RDW were standardized prior to analyses in order to compare effects across different variables by solving shifting or scaling issues. The mediation analyses include the following steps. First, linear regression was applied to examine the associations between sodium and CRP (or RDW), adjusting for age, sex, ethnicity, education level, family monthly poverty level, passive sedentary hours, smoking status, drinking status, total energy intake, DII, and serum creatinine (path α). Second, CRP (or RDW) was additionally controlled in the logistic regression model to estimate the direct effect of sodium intake on NAFLD, adjusting for the same covariates (path *γ*’). Third, the mediate function from the R mediation package with the causal analytical approach was applied to quantify the indirect effect (path *α* & path *β*) and the total effect (path *γ*) [26, 27]. To observe the effect of β-carotene on the mediating effects, we repeated the above analyses with adjustment for β-carotene. The mediation package is freely available for download via the Comprehensive R Archive Network (CRAN) at http://CRAN.R-project.org/package=mediation. A detailed description of the construction and statistic approaches for the mediation package is available at https://cran.r-project.org/web/packages/mediation/vignettes/mediation.pdf. The mediation analyses diagram for the association of dietary sodium and β-carotene intakes with NAFLD is shown in Fig. 2.

A two-tailed *P* < 0.05 was considered significant. Analyses were performed using SAS version 9.4 (SAS Institute, Cary, North Carolina) and R version 3.6.3 (R Foundation for Statistical Computing, Vienna, Austria).

## Results

A total of 6725 participants (3119 men and 3606 women) with a mean age of 53.2 ± 17.4 years were included in this study. The characteristics of the study population by the combination groups of dietary sodium and β-carotene intakes are presented in Table 1. Compared with participants with high sodium and low β-carotene intakes, those with high sodium and high β-carotene intakes were older and more likely to be white race, to have higher education level, to have higher family income, to have longer passive sedentary time, and to have lower DII and log-transformed CRP level. In addition, those with high sodium and high β-carotene intakes were more likely to be drinker and less likely to be current smoker.

**Table 1:** Characteristics of the study population by combinations of dietary sodium and β-carotene intakes (*n* = 6725).

Characteristics	Low Na-High carotene	Low Na-Low carotene	High Na-High carotene	High Na-Low carotene	*P*-values
*n*	1513	1847	1851	1514	
Age, years	59.0 ± 16.2	54.1 ± 17.9	52.2 ± 16.9^a^	47.4 ± 16.6	<0.0001
Men, *n* (%)	430 (28.4)	631 (34.2)	1104 (59.6)	954 (63.0)	<0.0001
Ethnicity, *n* (%)					<0.0001
Hispanic	403 (26.6)	573 (31.0)	394 (21.3)^a^	349 (23.1)	
White	808 (53.4)	855 (46.3)	1092 (59.0)^a^	805 (53.2)	
Black	254 (16.8)	370 (20.0)	258 (13.9)^a^	297 (19.6)	
Others	48 (3.2)	49 (2.7)	107 (5.8)^a^	63 (4.2)	
Education, *n* (%)					<0.0001
Less than high school	377 (24.9)	669 (36.2)	346 (18.7)^a^	351 (23.2)	
High school	351 (23.2)	461 (25.0)	329 (17.8)^a^	400 (26.4)	
More than high school	785 (51.9)	717 (38.8)	1176 (63.5)^a^	763 (50.4)	
Family monthly poverty levels					<0.0001
≤1.30	406 (26.8)	697 (37.7)	414 (22.4)^a^	473 (31.2)	
1.31–1.85	279 (18.4)	404 (21.9)	270 (14.6)^a^	267 (17.6)	
>1.85	828 (54.7)	746 (40.4)	1167 (63.1)^a^	774 (51.1)	
Smoking status, *n* (%)					<0.0001
Current	143 (9.5)	356 (19.3)	224 (12.1)^a^	317 (20.9)	
Former	400 (26.4)	462 (25.0)	582 (31.4)^a^	385 (25.4)	
Never	970 (64.1)	1029 (55.7)	1045 (56.5)^a^	812 (53.6)	
Drinker, *n* (%)	829 (54.8)	1009 (54.6)	1352 (73.0)^a^	1030 (68.0)	<0.0001
Hypertension, *n* (%)	718 (47.5)	819 (44.3)	686 (37.1)	530 (35.0)	<0.0001
Diabetes, *n* (%)	223 (14.7)	282 (15.3)	237 (12.8)	204 (13.5)	0.1298
Sedentary time, h/day	5.1 ± 3.2	5.0 ± 3.2	5.8 ± 3.5^a^	5.4 ± 3.2	<0.0001
Dietary inflammatory index	0.5 ± 1.3	1.5 ± 1.1	−0.9 ± 1.4^a^	0.1 ± 1.3	<0.0001
Total energy, kcal	1527 ± 402	1479 ± 423	2433 ± 741	2428 ± 689	<0.0001
Body mass index, kg/m^2^	28.5 ± 6.1	29.3 ± 6.6	29.0 ± 6.6^a^	29.9 ± 7.2	<0.0001
Log-transformed c-reactive protein, mg/dL	−1.7 ± 1.2	−1.5 ± 1.2	−1.8 ± 1.3^a^	−1.7 ± 1.3	<0.0001
Log-transformed red cell distribution width, %	2.6 ± 0.1	2.6 ± 0.1	2.5 ± 0.1	2.6 ± 0.1	<0.0001

Values are presented as mean ± standard deviation or *n* (%) unless otherwise indicated.

^a^Means there is significant difference compared with the group of high sodium-low carotene after Bonferroni adjustment.

Table 2 presents ORs and 95% CIs for the HSI and FLI defined NAFLD, respectively, by different combinations of dietary sodium and β-carotene intakes. Compared with the high sodium-low carotene group, participants in the high sodium-high carotene group had a lower odds for both the HSI and FLI defined NAFLD. The corresponding ORs and 95% CIs were 0.84 (0.73–0.98) for the HSI-defined NAFLD and 0.74 (0.60–0.92) for the FLI defined NAFLD after adjusting for age, sex, ethnicity, education level, family monthly poverty level, smoking, drinking, sedentary time, total energy intake, DII, and serum creatinine. Besides, participants in the low sodium-high carotene group had the lowest odds for NAFLD compared with the high sodium-low carotene group.

**Table 2:** Odds ratio (OR) and 95% confidence interval (CI) for NAFLD by the combinations of dietary sodium and β-carotene intakes.

	Low Na-High carotene	Low Na-Low carotene	High Na-High carotene	High Na-Low carotene
HSI-defined NAFLD				
*n*	1513	1847	1851	1514
No. of cases	814	1061	1003	922
Prevalence^a^, %	53.8	57.4	54.2	60.9
ORs (95% CIs)				
Model 1^b^	0.65 (0.56–0.76)	0.78 (0.68–0.90)	0.74 (0.64–0.85)	1 (Reference)
Model 2^c^	0.60 (0.51–0.71)	0.62 (0.53–0.73)	0.84 (0.73–0.98)	1 (Reference)
FLI-defined NAFLD				
*n*	727	891	892	727
No. of cases	284	391	373	376
Prevalence^d^, *n* (%)	39.1	43.9	41.8	51.7
ORs (95% CIs)				
Model 1^b^	0.54 (0.43–0.67)	0.71 (0.58–0.88)	0.64 (0.52–0.78)	1 (Reference)
Model 2^c^	0.53 (0.41–0.68)	0.58 (0.46–0.73)	0.74 (0.60–0.92)	1 (Reference)

^a^*P*-value for difference in terms of prevalence of NAFLD equals to 0.0001.

^b^Adjusted for age and sex.

^c^Adjusted for age, sex, ethnicity, education level, family monthly poverty level, smoking, drinking, sedentary time, total energy intake, dietary inflammatory index, and serum creatinine.

^d^*P*-value for difference in terms of prevalence of NAFLD < 0.0001. The median values used in the HSI analysis were 3032 mg/day for sodium intake and 1217.5 µg/day for β-carotene intake. The median values used in the FLI analysis were 3049.5 mg/day for sodium intake and 1235.5 µg/day for β-carotene intake.

**Fig. 3:**
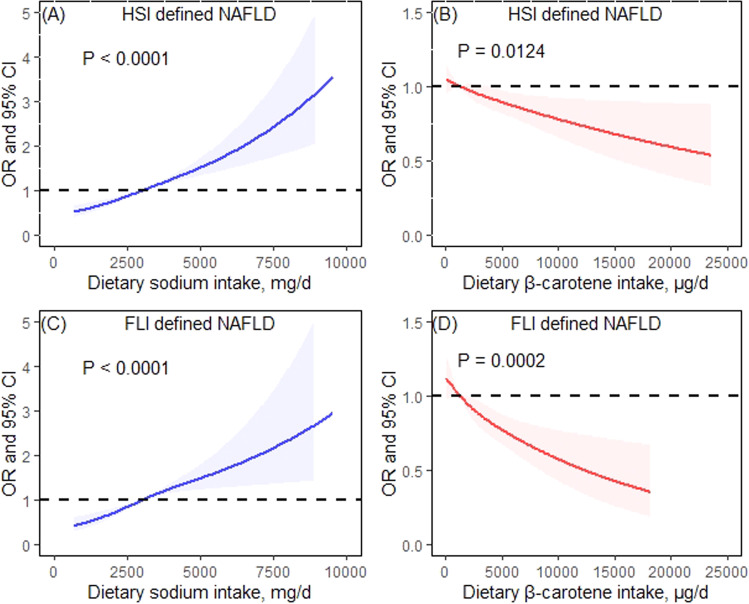
Dose–response associations of dietary sodium and β-carotene intakes with no-alcoholic fatty liver disease (NAFLD). The restricted cubic spline model was basically adjusted for age, sex, ethnicity, education level, family monthly poverty level, smoking, drinking, sedentary time, total energy intake, dietary inflammatory index, and serum creatinine. **A** and **C** additionally adjusted for dietary β-carotene intake; **B** and **D** additionally adjusted for dietary sodium intake.

The dose–response relations of dietary sodium and β-carotene intakes with NAFLD defined by HSI and FLI were shown in Fig. 3. There was a significant positive dose–response relationship between dietary sodium intake and NAFLD after adjusting for covariates described above plus β-carotene (*P* < 0.001). In contrast, there was a significant inverse association between dietary β-carotene intake and NAFLD after adjustment for covariates including dietary sodium intake (*P* < 0.05).

Results from the mediation analysis for the association between dietary sodium intake and NAFLD with or without adjustment for dietary β-carotene intake were shown in Table 3. There were positive indirect effects of dietary sodium intake on the HSI-defined NAFLD (indirect effect: 0.0057, 95% CI: 0.0021–0.0091, *P* < 0.0001) as well as the FLI defined NAFLD (indirect effect: 0.0081, 95% CI: 0.0024–0.0162, *P* < 0.0001) when CRP was considered as a mediator. As expected, the mediating effects were somewhat attenuated when we further adjusted for dietary β-carotene intake. Similar results were found when RDW was considered as a mediator in the HSI analysis. However, the mediating effect of RDW on the relationship between dietary sodium intake and FLI defined NAFLD did not reach the statistical significant level.

**Table 3:** Mediation effects of dietary sodium on NAFLD with CRP and red cell distribution width as mediators.

	Without adjustment for β-carotene			With adjustment for β-carotene		
	Estimates^a^	95% CIs	*P*-values	Estimates^b^	95% CIs	*P*-values
HSI-defined NAFLD						
CRP						
Total effect	0.0662	0.0497–0.0862	<0.0001	0.0683	0.0492–0.0844	<0.0001
Direct effect	0.0605	0.0402–0.0788	<0.0001	0.0633	0.0432–0.0775	<0.0001
Indirect effect	0.0057	0.0021–0.0091	<0.0001	0.0050	0.0021–0.0083	<0.0001
Red cell distribution width						
Total effect	0.0675	0.0483–0.0883	<0.0001	0.0676	0.0534–0.0826	<0.0001
Direct effect	0.0663	0.0478–0.0869	<0.0001	0.0665	0.0529–0.0819	<0.0001
Indirect effect	0.0012	0.0002–0.0027	<0.0001	0.0011	0.0002–0.0022	0.0400
FLI-defined NAFLD						
CRP						
Total effect	0.0686	0.0374–0.1010	<0.0001	0.0752	0.0481–0.1072	<0.0001
Direct effect	0.0605	0.0322–0.0918	<0.0001	0.0680	0.0402–0.0992	<0.0001
Indirect effect	0.0081	0.0024–0.0162	<0.0001	0.0072	0.0009–0.0121	<0.0001
Red cell distribution width						
Total effect	0.0701	0.0383–0.0981	<0.0001	0.0747	0.0501–0.1072	<0.0001
Direct effect	0.0692	0.0384–0.0967	<0.0001	0.0739	0.0490–0.1071	<0.0001
Indirect effect	0.0009	−0.0001 to 0.0027	0.0800	0.0008	−0.0004 to 0.0019	0.2000

*CI* confidence interval, *CRP* c-reactive protein, *FLI* fatty liver index, *HSI* hepatic steatosis index.

^a^Adjusted for age, sex, ethnicity, education level, family monthly poverty level, smoking, drinking, sedentary time, total energy intake, dietary inflammatory index, and serum creatinine.

^b^Adjusted for age, sex, ethnicity, education level, family monthly poverty level, smoking, drinking, sedentary time, total energy intake, dietary inflammatory index, serum creatinine, and dietary β-carotene.

## Discussion

In this study on a representative sample of US adults, we found that dietary sodium intake was positively associated with NAFLD, while dietary β-carotene intake was inversely associated with NAFLD. Participants who had high sodium and high β-carotene intakes had a lower odds of NAFLD compared with those had high sodium and low β-carotene intakes. The mediation analysis supports the association of dietary sodium with the NAFLD, being, at least partially, mediated by the effect of inflammation.

In recent years, several previous studies including our own study reported an independent relationship between dietary sodium intake and NAFLD. A study using data from the Korea National Health and Nutrition Examination Surveys (2008–2010) found significant associations between higher estimated 24-h urinary sodium excretion and NAFLD as assessed by both HSI (OR: 1.39, 95% CI: 1.26–1.55) and FLI (OR: 1.75, 95% CI: 1.39–2.20) [28]. Another study conducted in the Korea population also suggested that higher dietary sodium intake was associated with a greater prevalence of NAFLD in young and middle-aged general adults [29]. Results from the Prevention of Renal and Vascular End-Stage Disease (PREVEND) cohort study showed that higher dietary sodium intake was positively associated with NAFLD as defined by the FLI, with an OR per SD increment of 1.30 (95% CI: 1.21–1.41). Similar results were also shown for the HSI-defined NAFLD, where the corresponding OR and 95% CI were 1.40 (1.31–1.51) [30]. Our previous study based on NHANES found that comparing with the lowest quartile of dietary sodium intake, the highest quartile had a multivariable-adjusted OR and 95 % CI of 1.46 (1.29–1.65) for NAFLD as defined by HSI, and 1.41 (1.18–1.69) for NAFLD as defined by FLI [8]. The dose–response relationship between dietary sodium intake and NAFLD deprived from the restricted cubic regression model in the present study is in line with previous observational studies. However, the mechanism underlying this relationship remains unclear.

Carotenoids form an important part of the human diet, consumption of which has been associated with many health benefits. β-carotene is one of the main constituents of carotenoids [31]. A community-based cross-sectional study conducted in a Chinese population observed a dose-dependent inverse association between β-carotene and NAFLD. The OR and 95% CI of NAFLD for the highest quartile was 0.32 (0.25–0.41) compared with the lowest quartile [32]. A case–control study enrolled 24 control subjects and 62 NAFLD patients, whose liver biopsies were collected and histological characteristics were assessed. Compared with individuals without hepatic steatosis, those with moderate steatosis had significantly decreased serum β-carotene (0.34 ± 0.03 vs. 0.21 ± 0.02 μmol/L). Moreover, β-carotene gradually decreased along disease progression from normal liver, simple steatosis to borderline steatohepatitis (*P* for trend ≤0.001) [33]. A prospective cohort study enrolled 2687 subjects who completed both NAFLD tests were classified into stable, improved and progressed groups according to changes in the degree of NAFLD between two visits. Analysis of covariance showed that ln-transformed serum β-carotene was positively associated with NAFLD improvement (*P* for trend < 0.05). After multivariable adjustment, mean difference in serum β-carotene was higher by 29.6% in the improved vs. progressed subjects [34].

The pro-inflammatory effect of sodium has been reported in several previous studies. In animal experiments, high sodium diet triggers TH17 (interleukin-17 (IL-17)-producing helper T cells) development and promotes tissue inflammation. TH17 cells are highly pro-inflammatory cells that are critical for clearing extracellular pathogens and for inducing multiple autoimmune diseases [35–37]. Results from a recent study showed that mice fed with a high-salt diet displayed obvious hepatic steatosis and inflammation. All these pathological changes persisted after salt withdrawal, displaying a memory phenomenon [38]. In human studies, high sodium intake was reported to be associated with changes in inflammatory markers including tumor necrosis factor-α (TNF-α) and adiponectin [39–41]. Contrary to the pro-inflammatory effect of sodium, β-carotene has a strong anti-inflammatory effect [42]. A previous study suggested that β-carotene decreases the severity of C-C motif chemokine ligand 4 (CCL4) induced hepatic inflammation and fibrosis in rats [43]. Another study reported that tomato powder (rich in β-carotene) inhibits hepatic steatosis and inflammation potentially through restoring sirturin 1 (SIRT1) activity and adiponectin function [44].

To the best of our knowledge, this is the first study to explore the pro-inflammatory mechanism of higher sodium intake in relation to an increased odds of NAFLD in humans through a mediation analysis. Moreover, we also found that dietary β-carotene attenuates the mediating effects of inflammation on the association between dietary sodium intake and NAFLD. Our study has limitations: First, dietary sodium intake was measured from two 24-h dietary recalls instead of multiple 24-h urine collections. The latter is considered as the gold-standard for estimating sodium intake in humans. However, the lack of urinary sodium measurements in this study limits our efforts to use more objective sodium measurement indicators to verify the reliability of the results. Second, NAFLD was defined using HSI- and FLI- based predictive equations rather than hepatic biopsy. Although hepatic biopsy method has a better accuracy for the diagnosis of NAFLD, it is difficult to apply in large-scale population studies. Third, given the enormous complexity of the inflammatory response, CRP and RDW provide only limited information. Future research should thus focus on additional biomarkers, such as IL-1β, IL-6, TNF-α, and CD4 + T cell subsets. Lastly, the cross-sectional study design is subject to recall bias and this design cannot prove causality in itself. Findings from our study should be further confirmed in well-designed prospective cohort studies using more reliable sodium measurements in future.

## Conclusions

This study suggests that higher sodium intake increases the odds of NAFLD by upregulating inflammation. Dietary β-carotene may attenuate this association by down regulating inflammation.

## Data Availability

All data used in this manuscript are de-identified and freely available to the public through https://www.cdc.gov/nchs/nhanes/
